# Central vs. Peripheral Action of Thyroid Hormone in Adaptive Thermogenesis: A Burning Topic

**DOI:** 10.3390/cells10061327

**Published:** 2021-05-27

**Authors:** Yanis Zekri, Frédéric Flamant, Karine Gauthier

**Affiliations:** Institut de Génomique Fonctionnelle de Lyon, Univ Lyon, CNRS UMR 5242, INRAE USC 1370 École Normale Supérieure de Lyon, Université Claude Bernard Lyon 1, 46 allée d’Italie, 69007 Lyon, France; frederic.flamant@ens-lyon.fr (F.F.); karine.gauthier-vanacker@ens-lyon.fr (K.G.)

**Keywords:** thyroid hormones, thermogenesis, brown adipose tissue, browning, hypothalamus

## Abstract

Thyroid hormones (TH) contribute to the control of adaptive thermogenesis, which is associated with both higher energy expenditure and lower body mass index. While it was clearly established that TH act directly in the target tissues to fulfill its metabolic activities, some studies have rather suggested that TH act in the hypothalamus to control these processes. This paradigm shift has subjected the topic to intense debates. This review aims to recapitulate how TH control adaptive thermogenesis and to what extent the brain is involved in this process. This is of crucial importance for the design of new pharmacological agents that would take advantage of the TH metabolic properties.

## 1. Introduction

Obesity is an uncontrolled worldwide pandemic whose incidence has tripled during the last forty years. Given its social and economic burden as well as the abundance of its comorbidities such as diabetes, hypertension or atherosclerosis, many studies aimed at isolating pharmacological targets to prevent and fight this condition. Among the numerous therapeutic possibilities, thyroid hormones (TH, including thyroxine, or T4, and tri-iodo-thyronine, or T3, its more active metabolite) emerged as promising candidates. Already in 1895, Adolf Magnus Levy reported the influence of the thyroid status on the human basal metabolic rate. It was confirmed later that the level of circulating T3 is correlated with energy expenditure in humans [1,2]: hypothyroidism and hyperthyroidism are respectively associated with low and high energy expenditure but most importantly to high and low body mass index [3]. Similar effects are observed with exogenous T3 treatment in mice [4]. However, T3 cannot be used as a pharmacological agent since it also triggers tachycardia, lean mass loss and osteoporosis [5,6,7]. Thus, a recent intense effort has been dedicated to understanding how T3 fulfills its different metabolic activities, looking for the target tissues and the specific thyroid hormone receptors (TRs) involved. The final goal would be to identify new chemical compounds that could uncouple the metabolic benefits of T3 from its adverse effects.

The action of T3 on energy expenditure is traditionally considered to result from its local action in several metabolic tissues. However, an alternative possibility is that T3 acts in the hypothalamus, setting the sympathetic tune and stimulating the activity of distant tissues [8]. In both hypotheses, T3 acts via its binding to local TRs, either in metabolic tissue or hypothalamus of which respective importance remain controversial. It is yet crucial for the development of new pharmacological reagents which aim to stimulate T3 signaling in a tissue-selective manner to increase energy expenditure.

## 2. Energy Expenditure and Adipose Tissues: The Main Route for Adaptive Thermogenesis

### 2.1. Adaptive Thermogenesis as a Way to Modulate Energy Expenditure

Energy expenditure is defined as a combination of both basal and adaptive thermogenesis [9]. Basal thermogenesis is the heat produced by a resting organism through all its basal exothermic metabolic processes. Thermoneutrality is then defined as the range of ambient temperature within which the body temperature can be maintained only relying on heat produced by the biochemical transformations occurring at basal metabolic rate [10]. In contrast, adaptive thermogenesis corresponds to the extra-heat produced by a combination of physical activity and specific responses triggered to face physiological stressors, including diet or ambient temperatures below thermoneutrality [9]. Thus, adaptive thermogenesis is a tunable component of a particular interest to increase energy expenditure. It can occur through shivering, involving the muscles, or to a greater extent through non-shivering mechanisms in which both adipose tissues and muscles contribute. The regulation of energy expenditure involves a dialogue between the autonomous nervous system and major peripheral organs such as the liver, the heart, the muscles, the white adipose tissue (WAT), and the brown adipose tissue (BAT). These peripheral organs also communicate together by releasing in the circulation several diffusible factors [11,12,13,14,15]. 

### 2.2. Adipose Tissues and Muscle, the Main Actors of Adaptive Thermogenesis

**Figure 1:**
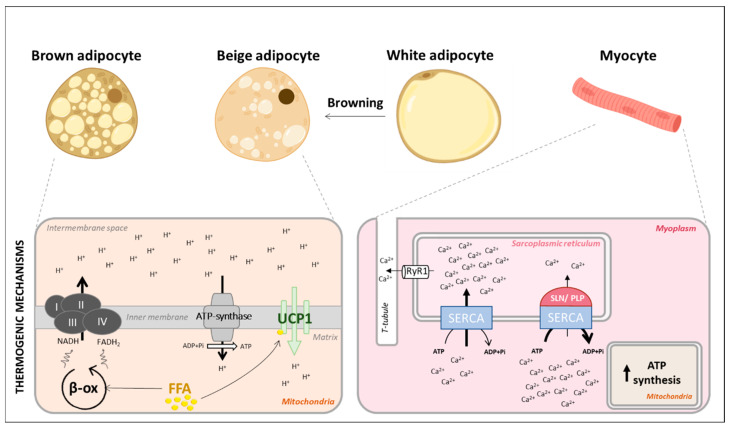
The main actors of adaptive thermogenesis and their principle thermogenic mechanisms. Adipose tissues and muscles are the main actors of adaptive thermogenesis. White adipocytes are not thermogenic per se but can undergo browning to generate beige adipocytes. *Bottom left panel:* Brown and beige adipocytes use free fatty acids (FFA) to fuel the mitochondrial β-oxidation. β-oxidation generates reduced compounds (NADH, FADH_2_) whose oxidation is used by the respiratory electron transport chain (complex I, II, III, IV) to pump protons (H^+^) into the intermembrane space. Thus, an electrochemical gradient is created and used by ATP synthase to produce ATP. UCP1 is present in the inner mitochondrial membrane and activated by FFA. UCP1 acts as a proton channel to dissipate the electrochemical gradient without producing ATP. Thus, to match the inefficient ATP production, the metabolism must increase and heat is produced. *Bottom right panel:* Myocytes express SERCA that is located in the sarcoplasmic reticulum (SR) membrane. SERCA transfers calcium (Ca^2+^) from the cytosol to SR lumen using ATP hydrolysis. The calcium gradient generated by SERCA is dissipated by ryanodine receptor (RyR1). SERCA transport activity can be inhibited by two peptides: phospholamban (PLP) or sarcolipin (SLN), but its ATPase activity remains. To match Ca^2+^ transport, ATP mitochondrial synthesis increases and heat is produced.

The three different types of adipose tissues are defined by their cell types composition, location, and subsequent functionality. Brown adipose tissue (BAT) is mainly composed of brown adipocytes characterized by a high mitochondrial content, generating its distinctive color, and giving this tissue a high respiration potential [16,17,18]. Brown adipocytes produce the uncoupling protein 1, UCP1, an inner mitochondrial membrane protein which, on activation by free fatty acids, drives the uncoupling of oxidative phosphorylation from ATP production by operating as a proton carrier (Figure 1). The lipid catabolism by brown adipocytes thus does not result in ATP production but in exothermic reactions [19,20]. Lipids, the main fuel of BAT thermogenesis, mainly come from brown adipocyte lipid droplets but can also be imported from circulation [21,22]. Glucose metabolism is also crucial for BAT activity as it contributes to lipogenesis to replenish lipid droplets or can be used as an alternative fuel [23,24,25,26]. However, while UCP1 was historically considered the only crucial mediator of BAT thermogenesis, recent work suggests that other mechanisms also participate to this process. For instance, adipocyte-specific deletion of the mitochondrial creatine kinase b (*Ckb*) markedly decreases the thermogenic response after β3-adrenergic receptor stimulation [27]. CKB triggers ATP-dependent creatine phosphorylation, concomitantly with the activity of phosphatases that dephosphorylate it, generating a futile cycle [28]. This mechanism contributes to energy expenditure even in the presence of UCP1 [27]. It clearly highlights that there are UCP1-independent BAT thermogenic mechanisms [20,29], with more to be discovered. 

On the contrary, adipocytes of the white adipose tissue (WAT) have reduced metabolic activity. They ensure lipid storage, favoring lipogenesis during calorie excess and breaking down triglycerides during energy restriction to fuel other organs’ activity [30]. Thus, white adipocytes are not able of adaptive thermogenesis. However, upon prolonged cold exposure, beige adipocytes emerge in WAT depots, a process known as browning, or beiging [31,32]. Beige adipocytes express *Ucp1* [33] and other thermogenic markers [34], granting them the capacity to spend energy through thermogenesis (Figure 1).

In humans, BAT is mainly found in infants. In adults, browning can take place under certain conditions within specific WAT depots, particularly in the abdominal, paraspinal, supraclavicular and cervical regions [35]. Transcriptome analyses confirmed that these *Ucp1* expressing cells are more closely related to rodent beige adipocytes than to brown adipocytes [36,37]. Browning in adults can be triggered [38,39,40,41], which could be promising in the treatment of obesity. However, the significance of beige fat contribution on energy expenditure is still a matter of debate [42,43,44]. 

Muscle is also a thermogenic organ. It increases energy expenditure by inducing the sarcoplasmic and endoplasmic Ca^2+^-dependent ATPase (SERCA) that breaks down ATP to transport Ca^2+^ from cytosol to reticulum lumen. SERCA activity is regulated by two peptides that uncouple Ca^2+^ transport to ATP breakdown (Figure 1), generating a futile cycle and ultimately heat production [45,46,47,48]. Muscle cells also produce UCP3, an UCP1- related protein which uncoupling capacity remains unclear [49,50]. As skeletal muscle is reckoned to represent as around 40% of the total body mass, we can expect that only minor changes in its non-shivering thermogenesis could largely contribute to whole-body thermogenesis and energy expenditure.

### 2.3. Adaptive Thermogenesis Is Induced by Cold Exposure and High Fat Diet

**Figure 2:**
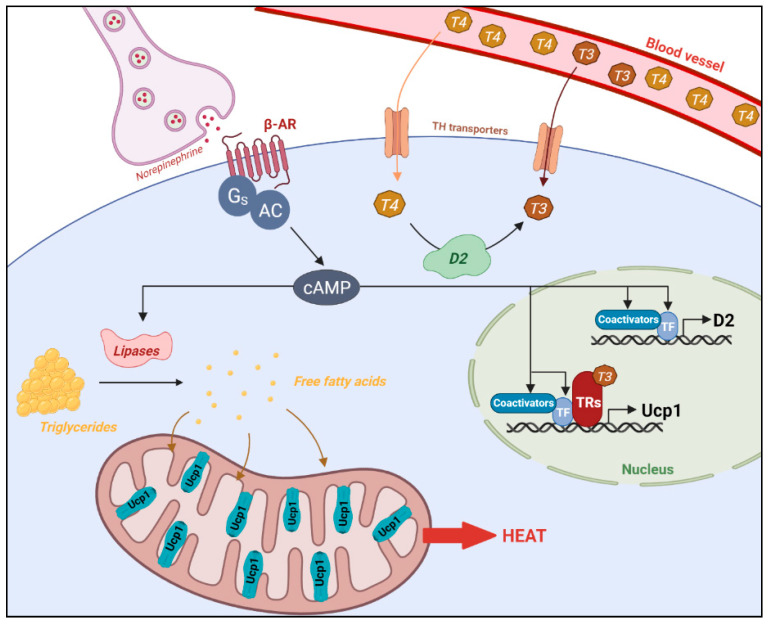
Control of brown adipocyte UCP1-dependent thermogenesis by norepinephrine and thyroid hormone. Sympathetic neurons release synaptic norepinephrine that binds to β-adrenergic receptors (β-AR) coupled to stimulatory guanine nucleotide binding protein (Gs) which activates adenylate cyclase (AC) to produce cAMP. This adrenergic signaling activates transcription factors (TF) and coactivators involved in the regulation of *D2*. Both adrenergic signaling and thyroid hormone receptors (TRs) regulate *Ucp1* expression. Triglycerides are broken down into free fatty acids by lipases and transported to mitochondria to fuel the β-oxidation and activate UCP1. UCP1 uncouples ATP production from respiration, requiring an increased mitochondrial activity and heat is produced.

Adaptive thermogenesis can be triggered by two natural drivers: cold exposure [16,51] that increases the demand of heat production to maintain the body temperature, and high fat diet [52,53,54,55] that stimulates the elimination of excessive calories. Cold is sensed by thermoreceptors in the cutaneous terminals of primary somatosensory neurons [56,57]. High fat diet signaling likely involves the cholecystokinin release from endocrine cells of the small intestine, which triggers excitation of gut vagal afferents [58,59]. In both cases, the stress signal is integrated by the hypothalamus that rapidly triggers the release of norepinephrine (NE) from the nerve terminals of the sympathetic nervous system (SNS) innervating the BAT, and stimulating the β-adrenergic receptors present at the surface of the adipocytes. The signal is then relayed intracellularly by the cAMP-dependent protein kinase A (Figure 2) [16]. Additionally, while cold exposure provokes WAT browning [26,60,61], the effects of high fat diet on this process are still conflicting [62,63]. As expected, the concomitant knock-out of the 3 β-adrenergic receptors (β-AR) leads to cold hypersensitivity [64] and to an increased sensitivity to diet-induced obesity at thermoneutrality [65]. A similar phenotype is observed in *Ucp1KO* mice [66,67], which historically designated UCP1 as the only crucial mediator of BAT thermogenesis. However, *Ucp1KO* animals display altered mitochondrial respiration and are more susceptible to reactive oxygen species [68]. In the absence of functional mitochondria, any UCP1-independent mechanism involving mitochondria would not be efficient in the *Ucp1KO* mice and thus difficult to unravel. Thus, the below-mentioned papers mainly concluding on the role of UCP1 should not be overinterpreted. Some other mechanisms might be involved, as previously mentioned for creatine futile cycles [27,28].

Interestingly, thyroid hormones status alters the thermogenesis in response to both cold [69,70,71] and high fat diet [72], pointing out that they are a crucial component when it comes to regulate energy expenditure and adaptive thermogenesis.

## 3. T3, an Important Component of Energy Expenditure

The thyroid gland mainly produces T4, the inactive form of thyroid hormone, which is converted to active T3 by deiodination in other organs [73]. While the serum levels of T4 and T3 are normally maintained in a narrow range, local deiodination can increase the T3 level by the type 2 deiodinase (D2) in different organs. T3 signaling is mediated by nuclear receptors TRα1, TRβ1 and TRβ2 (collectively TRs) produced by *Thra* and *Thrb*, two genes which are expressed in many cell types [74,75]. These receptors are bound to specific DNA sequences and either repress or activate the transcription of neighboring genes depending on T3 binding [76,77,78].

### 3.1. T3 Signaling Is Necessary for Cold-Induced Thermogenesis

*D2* is expressed in BAT (Figure 2) and its activity can be locally induced upon cold exposure [79], triggering within a few hours a fivefold increase in the local concentration of T3 [80]. It leads to an increased TR activity within 24 h [81]. Accordingly, *D2KO* mice that lack D2 activity [82], are more sensitive to cold than WT mice, losing more weight and failing in efficiently defend their core body temperature when placed at 4 °C [83]. Their BAT does not fully respond to SNS stimulation. Importantly, brown adipocytes isolated from *D2KO* mice also fail to efficiently increase oxygen consumption and cAMP accumulation upon norepinephrine stimulation. Therefore, β-AR sensitivity is highly dependent on T3 produced in BAT. Moreover, the dysfunctional adaptive thermogenesis observed in vivo is not due to a lack of UCP1 activation or a defective lipolysis, but from impaired lipogenesis, which disrupts the restoration of BAT lipids [84]. To compensate for the altered thermogenesis, *D2KO* mice mount an exaggerated SNS response below thermoneutrality. The permanent stimulation of BAT leads to an overexpression of the *Ucp1* gene and to an increased lipolysis. The unopposed and persistent stimulation in absence of lipogenesis results in the exhaustion of the free fatty acids storage in brown adipocytes, preventing an efficient BAT thermogenesis and ultimately leading to hypothermia. This suggests that in addition to increasing the sensitivity of BAT to β-AR, T3 also induces lipogenesis in brown adipocytes. 

### 3.2. The Role of T3 Signaling in Response to Diet-Induced Obesity

Thermogenesis is also triggered by an excess of circulating lipids after a high fat diet. The role of T3 signaling in this process is less documented. At 23 °C, *D2KO* mice develop obesity similarly to control in response to a high fat diet [85]. As 23 °C is below thermoneutrality and represents a mild cold stress [86], the SNS outflow increases to raise energy expenditure, independently of T3. As mentioned above, in *D2KO* mice, this activation is even higher to bypass a decrease in SNS sensitivity. Thus, it prevents obesity despite a lack of D2. However, *D2KO* mice become hypersensitive to obesity at 30 °C [85], a temperature at which SNS does not stimulate BAT thermogenesis. Consequently, there is no compensation for the lack of local T3 that must be needed in these conditions to increase energy expenditure and limit weight gain. This is accompanied by a blunted response of *Ucp1* expression in the BAT [85]. This suggests that a local increase of T3 catalyzed by D2 is important to increase energy expenditure in BAT after a high fat diet. Mice KO for A-FABP, an adipokine fatty acid-binding protein, fail to induce *D2* expression in the BAT. Subsequently, these mice cannot respond to either cold or high fat diet [87], supporting the crucial role of T3 in these two responses.

### 3.3. Tissue-Selective Metabolic Action for T3 Signaling

The different studies cited and analyzed above clearly establish that T3 signaling is critical to trigger adaptive thermogenesis both in response to cold and to a high fat diet and that *D2* up-regulation in BAT is likely to be involved in both cases. *D2* is expressed in the BAT [88] but also in myotubes [89,90,91]. To address the respective contribution of adipocytes and muscle fibers in energy metabolism, tissue-selective knock-out (KO) were performed. The consequences of inactivating D2 from *Fabp4*-expressing white and brown adipocytes (*FAT-D2KO*) or *Myosin light-chain 1f*-expressing skeletal muscle fibers (*SM-D2KO*) have been compared to a general KO of this enzyme (*GLOB-D2KO*) [92].

*SM-D2KO* mice do respond normally to cold or high fat diet. This suggests a negligible contribution of T3 produced in myotubes to adaptive thermogenesis. Absence of D2 would rather lead to changes in muscle contractile functions and fiber type composition [93].

Unlike *GLOB-D2KO* mice, *FAT-D2KO* mice are hypersensitive to diet-induced obesity at 23 °C. *FAT-D2KO* mice have a reduced contribution of fatty acids to energy expenditure and mainly use glucose as a source of energy. This phenotype indicates that locally produced T3 accelerates fatty acids oxidation in BAT, a process required for BAT activation and optimal *Ucp1* expression/activity [94]. It can be hypothesized that the increase in glucose oxidation in *FAT-D2KO* mice is a compensation for the altered fatty acids oxidation, but does not produce as much heat in response to a high fat diet [95]. Altered fatty acids oxidation could therefore explain the higher weight gain. 

### 3.4. TR Isoform Selective Regulation of Adaptive Thermogenesis

Many mice models with knock-out (KO) or knock-in (KI) mutations of TRs have been generated [96] to elicit the role of T3 action and the TR isoform specificity on energy expenditure and thermogenesis. *Thra/Thrb* KO mice, devoid of all receptors, have a lower body temperature at thermoneutrality [97,98] and fail to defend their temperature when exposed to cold [98]. *Thra* KO mice, whose TRα1-expressing locus is deleted [97], also display a limited capacity for adaptive thermogenesis and display a profound hypothermia at 4 °C [99]. However, *Ucp1* expression is not down-regulated in *Thra* KO mice. This suggests that TRα1 function is not directly linked to the transcriptional regulation of *Ucp1*. Brown adipocytes cultivated from *Thra* KO mice do not respond to norepinephrine stimulation by an increased oxygen consumption, but the response of *Ucp1* and *D2* is maintained. KI mice, heterozygous for a dominant-negative mutated form of TRβ1 that cannot bind T3 [100], also have a defective thermogenesis but characterized by a reduction of *Ucp1* level and heat production during norepinephrine infusion [101]. Importantly, while isolated adipocytes from hypothyroid mice supplemented with T3 can induce cAMP production (reflecting the adrenergic responsiveness), they do not when supplemented with GC-1 [101], a selective TRβ agonist [102]. This emphasizes the importance of TRα1 in the brown adipocytes response. Altogether, the data suggest that the two receptors account for a specific subset of thermogenic function: TRβ1 is rather involved in the T3 regulation of *Ucp1* in BAT while TRα1 accounts for the sympathetic nervous system sensitivity.

## 4. Central T3 Can Trigger Adaptive Thermogenesis: The Still Controversial Role of the Brain

### 4.1. Role of Central T3 in the Activation of BAT Thermogenesis

**Figure 3:**
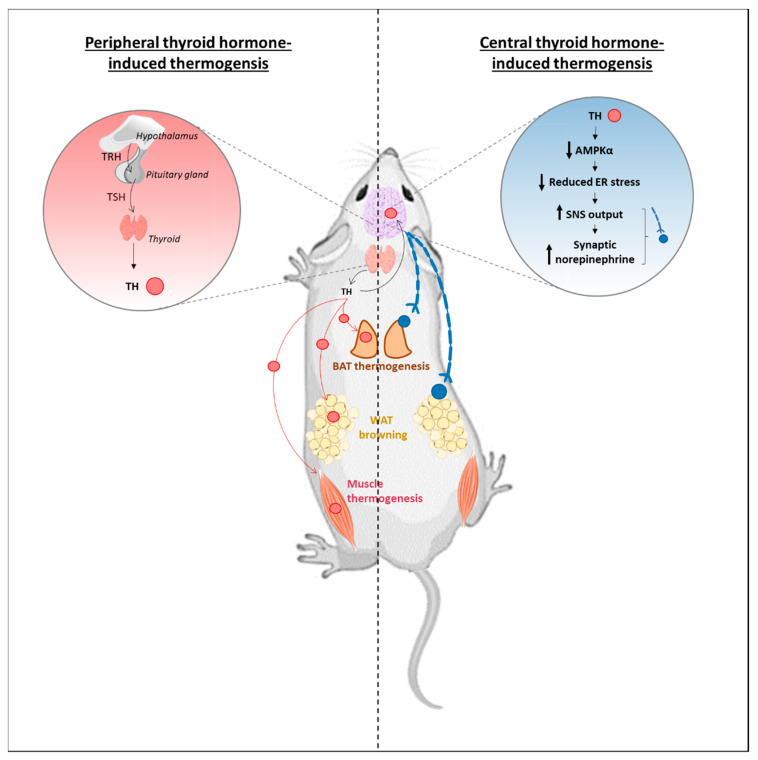
The different views for TH-induced adaptive thermogenesis. In the classical « peripheral » view (*left panel*), TH (red dots) are produced by the hypothalamic-pituitary-thyroid axis. TH are then released in the blood (red arrows) and are transported to targeted tissues. Then, they locally act on their receptors to trigger BAT and muscle thermogenesis, as well as WAT browning. This paradigm has been challenged by the description of a central mode of TH action to trigger adaptive thermogenesis (*right panel*). In this view, TH reaching the ventromedial medial hypothalamus decreases AMPKα phosphorylation in this region, alleviating endoplasmic reticulum (ER) stress. It leads to an increased sympathetic nervous system (SNS) output (axons drawn in blue) and the release of synaptic norepinephrine (blue dots) to trigger both BAT thermogenesis and WAT browning. However, no evidence as so far been brought to consider a TH-central control of muscle adaptive thermogenesis.

Based on the aforementioned role of BAT and the importance of *D2* expression and activity in this tissue, the classical view was that the thermogenic effect of T4/T3 mainly involves their direct action in the BAT (Figure 3). However, as early as in 1997, some authors already hypothesized that the thermal setpoint was centrally regulated and the effect of hyperthyroidism on BAT was the consequence of hyperthyroidism in the brain [103]. It was already known that electrical stimulation of the ventromedial hypothalamus (VMH) increases BAT temperature while VMH lesions inhibit thermogenesis [104,105]. As TRs and TH transporters are expressed in the VMH, TH signaling might be important in this brain area for the regulation of peripheral metabolism [106,107,108]. In 2010, Lopez et al. brought clear evidence that injection of T3 in VMH, but not in other hypothalamic nuclei, triggers a thermogenic response in BAT, an increase in energy expenditure and a subsequent weight loss without affecting food intake [8]. This treatment reduces hypothalamic AMPKα phosphorylation in the VMH, which in turn induces the SNS/β-AR system and activates the BAT (Figure 3), an effect that depends on TRs expression in the VMH. Both TRα and TRβ are present in the VMH; however, VMH-specific deletion of TRβ does not alter *Ucp1* BAT expression [109], suggesting that TRα in the VMH is the main contributor for the T3 centrally mediated BAT activation. 

Regulation of hypothalamic AMPKα activity to stimulate SNS and BAT thermogenesis has already been described for several hypothalamic peptides and hormones, including Bmp8b [110], Glp1 [111], estradiol [112] or leptin [113]. Recent work suggests that reduced AMPKα lowers endoplasmic reticulum stress in the hypothalamus [114] which in return facilitates BAT thermogenesis [115]. It is conceivable that central T3 uses this pathway to trigger its metabolic effects. However, the precise molecular mechanisms hidden behind remain unknown. 

Despite raising new possibilities and a fresher view for T3 mode of action, such studies are nevertheless controversial. Indeed, the implantation of bee wax pellets containing T3 in the VMH [116] does not reproduce the thermogenic response observed after VMH T3 injection [117]. In addition, T3 injected in the blood triggers a thermogenic response even in β-AR triple KO mice [118], arguing against a possible involvement of a SNS input to the BAT. *ASTRO-D2KO* mice that lack *D2* expression in *Gfap*-expressing astrocytes, display an increased BAT activity and an accelerated fatty acid oxidation [92]. As astrocytes are the only T3-producing cells in the brain [119], it emphasizes a role for central T3 in BAT activity. However, these observations are at odd with Lopez et al. conclusions [8] as it suggests that central T3 does not stimulate but rather slows down BAT thermogenesis. 

Interestingly, the thermogenic effects of injecting T3 in the VMH are observed in mice first exposed at 18 °C to recruit the BAT and then acclimated at 30 °C [8]. On the contrary, mice implanted with bee wax pellets, where no thermogenic effect of central T3 is observed, are kept at 23 °C. Yet, temperature is determinant when looking at BAT activity. As mentioned before, room temperature represents a mild cold stress exposure and activates BAT whereas thermoneutrality is obtained at 30 °C. In experiments using bee wax pellets, housing mice at 23 °C could constitutively activate BAT thermogenesis thus preventing any visible effect of central T3 on this process.

### 4.2. The Promising Metabolic Effects of WAT Browning: Also Concerned by a Central T3 Control?

In mice, browning can be triggered by three different mechanisms: (1) recruitment and activation of immune cells in WAT delivering norepinephrine locally [120], (2) direct action of hormones on white adipocytes, (3) SNS activation of the WAT. Some convincing elements have linked T3 to the two latter mechanisms. 

Rodents treatment with GC-1, a TR-β agonist, induces browning of subcutaneous WAT, increases energy expenditure, oxygen consumption, food intake and adiposity in both WT [121] and *ob*/*ob* obese mice [122]. GC-1 also reduces BAT activity, as testified by the decrease in *Ucp1* expression and the lower [^18^F]-FDG uptake in this tissue, which suggests that BAT is not responsible for the observed effects. Recently, Johann et al. showed that T3-induced WAT browning is neither associated with norepinephrine nor cAMP increase in the inguinal WAT. Moreover, browning is still observed at thermoneutrality, i.e., when the WAT is functionally denervated [123]. This is confirmed by the in vitro browning of primary white adipocytes treated with GC-1 [122]. This suggests that T3 induces browning through a peripheral mechanism. 

However, like BAT activity, WAT browning might also be sensitive to a central action of T3. T3 injection in the VMH, but not other brain regions, triggers WAT browning via the same AMPK-dependent pathway described for T3 mediated activation of the BAT [124]. These results reinforce other observations where T3 centrally administered by osmotic minipumps also increases WAT browning [125]. This common activation for both BAT and WAT could find its origin in shared neuronal pathways for BAT and WAT innervation [126,127]. 

Paradoxically, WAT browning is also observed in hypothyroid mice [128]. This is concomitant to a decrease in BAT activity despite a paradoxical high expression of thermogenic genes and norepinephrine concentration in this tissue. This likely points out to a primary defect in BAT thermogenesis, compensated by an increased sympathetic outflow to both BAT and WAT. A similar observation was already made in previous cold exposure studies [83,84]. 

Browning has raised many therapeutic interests in recent years, especially since beige adipocytes are a main site of adaptive thermogenesis in humans after cold exposure [40] and administration of β3-adrenergic receptor agonists [39,129]. Some results indicate that T3 might also potentiate browning in humans. First, D2 allowing conversion from T4 to T3, is expressed and active in human preadipocytes from both mesenteric and subcutaneous adipose tissues [130]. In addition, T3 also induces *Ucp1* expression, mitogenesis and oxygen consumption in a TRβ-dependent manner in vitro in multipotent adipose-derived stem cells [131]. In agreement, T3 has also recently been shown to induce *Ucp1* expression and reduce lipid accumulation in human white adipocytes [132]. This is in line with the observation that T4 serum levels in healthy subject correlates with *Ucp1* expression in WAT [124]. Taken together, these observations suggest that the T3 effect on human WAT browning might participate in the thermogenic response orchestrated by T3 injection. 

In addition, the thermogenic and metabolic responses to T3 are also observed in *Ucp1KO* animals [123,133]. This persistent response suggests that T3 triggers UCP1-independent thermogenic processes in brown/beige adipose tissues. It seems however that known mechanisms such as calcium and creatine are not inducible by T3 [28,134,135]. An alternative would be that T3-mediated thermogenic effects could also involve other tissues.

### 4.3. Muscle: Another Thermogenic Actor, Same Conflict?

Interestingly, it was observed that T3-mediated increase in body temperature in *Ucp1KO* mice is associated with a higher lipid uptake and a decrease in glycogen content in muscles [123]. In humans, increased TH levels trigger a higher glucose uptake in skeletal muscle than in BAT and WAT [136]. Increased metabolic rate is also noticed in the muscle of patients with THRB mutations that have high circulating levels of T4 and T3 [137]. As TRα1 is highly expressed in muscles [75], T3 might directly stimulates T3 responsive genes expression in this tissue.

Accordingly, T3 may increase muscle metabolic response. This has been assessed in a mouse model carrying a dominant-negative mutant TRα1 in *α-skeletal actin*-expressing cells [138], restricting the mutation to skeletal muscle and no other tissues [139]. This mutated form of TRα1 cannot recruit co-activators, preventing the T3-induced transcriptional response [140]. As expected, the increase of energy expenditure usually triggered by T3 is blunted in these mutant mice [138]. Muscles from mutants also display lower respiratory capacities ex vivo following T3 treatment. More surprisingly, unchallenged mutant mice display a paradoxical 5-fold increase of the muscle content in sarcolipin. This protein interacts with SERCA [141] to favor Ca^2+^ uncoupling from ATP hydrolysis (Figure 1), and thus contributes to the non-shivering thermogenesis in skeletal muscle [142]. The sarcolipin excess might thus represent a compensation for inadequate T3 response of the muscle thermogenesis. Neither muscle-specific deletion of D2 nor TRα1 dominant-negative expression alters energy expenditure or weight gain under high fat diet compared to littermate controls [92,138,143]. Collectively, it suggests that under a physiological stress, T3 in muscle might not be critical for its thermogenic action. It rather seems crucial for the response to pharmacological doses of T3. Finally, T3 intracerebroventricular injections does not induce thermogenic markers in muscle, reinforcing the idea that there is no indirect activation via the SNS [8] and that the metabolic effects mediated by T3 in muscle involve a local action.

## 5. Roles for T3 Central Action in Regulating Other SNS-Sensitive Mechanisms?

T3 and β-adrenergic signaling also regulate a common set of other physiological parameters. Following Lopez et al. observations [8], the question arose whether some of these SNS-sensitive mechanisms, which are not directly related to thermogenesis, could be regulated via a central action of T3. 

### 5.1. T3 Regulation of Glucose Homeostasis Is a Composite Process

Hypothyroidism is associated with a reduction in glucose uptake leading to peripheral insulin resistance, while hyperthyroidism increases hepatic gluconeogenesis and thus glycemia [144,145,146]. At least part of these effects is mediated through the direct binding of T3 on TRβ in the promoters of target genes [144,147].

As for BAT thermogenic action of T3, some evidence suggest that T3 central action could also be involved in regulating glucose metabolism. Indeed, mild hyperthyroidism increases glycemia and sympathetic liver denervation slightly prevents it. It suggests that the peripheral effects outweighed the central ones: central-T3 would rather fine-tuned the glucose production rather than hold the leadership. However, parasympathetic liver denervation worsens insulin resistance during T4 administration [148]. Work from the same group shows that T3 injection in the hypothalamic paraventricular nucleus (PVN) rapidly triggers endogenous glucose production without affecting circulating T3, an effect blunted after sympathetic denervation [149]. However, chronic PVN administration of T3 using bee wax pellets fails to recapitulate this effect as it did for BAT thermogenesis [117]. Collectively, these data argue for at least an involvement of central T3 in the regulation of glucose homeostasis.

### 5.2. T3 Regulation of Heart Rate and Hypertrophy

Heart rate is regulated by both T3 and the β-AR system. However, T3 cardiac action does not require β-AR since the T3-induced tachycardia and cardiac hypertrophy is intact in β-AR triple KO [118]. Moreover, many cardiac genes are directly regulated by T3 [150] and possess bona fide TR response element in their promoters [151]. This is in line with heart-specific *D2* overexpression that leads to tachycardia [152]. Finally, TRα1^+/m^ mice that express a mutant TRα1 with 10-fold reduced affinity to T3 [153], have a lower heart rate despite presenting hyperstimulation of the SNS [154].

Only few elements could indicate a central T3 control of heart processes. Indeed, Goldman et al. showed twenty years ago that short term intracerebral injection of T3 could stimulate heart rate [155]. Similarly, heart-specific *D2* overexpression fails to induce hypertrophy, suggesting a different mode of T3 control than for heart rate [152].

Collectively, these results argue mainly for a local, SNS-independent role of T3 in the heart. TRα1 is certainly the main mediator of this effect as it is the main isoform present in this tissue [75,156]. TRβ PV mutants that cannot bind T3, have shown to decrease heart rate and contractility, but without displaying mRNA changes [157].

## 6. Selective Mice Models and Pharmacology: New Perspectives to Better Understand and Take Advantage of T3 Metabolic Effects

### 6.1. Requirements for Elaborated Transgenic Models

Many effects mediated by T3 on adaptive thermogenesis have been deduced from the phenotype of transgenic mice models, mainly mutated for TRs and deiodinases. However, these results should be considered with caution due to these models’ spatiotemporal limitations. First, most of these models exhibit the mutations without tissue specificity, while deiodinases and TRs are widely expressed [75,158]. Thus, when the phenotype of a particular tissue is observed, it should be taken into consideration that it may indirectly results from alterations in other tissues reached by the mutation. In that respect, Cre-lox system became increasingly important to investigate the role of genes in a specific tissue/cell type [159]. For instance, tissue-specific deletion of *D2* has allowed to allocate the effects observed in *GLOB-D2KO* mice on lipid metabolism [85] to both astrocytes and BAT, but not muscle [92]. 

Secondly, most of these models harbor mutations from early stages of development while TH are crucial for many developmental processes [160,161,162]. Thus, the observations made on these models are limited as we cannot distinguish functional from developmental alterations. Notably, *D2KO* embryos have an altered BAT adipogenesis as well as thermogenic markers expression [163]. Thus, altered adaptive thermogenesis observed in the *D2KO* adults could stem from an inappropriate BAT maturation. It could be explained by the crucial role of T3 for the induction of lipogenesis [164], a process required for BAT growth [165]. In that respect, inducible models [166] allow triggering mutations at adult stages and therefore free observations from putative developmental defects.

It is thus of a particular interest to combine both Cre-lox and inducible systems [159]. For instance, specific promoter-induced expression of Cre recombinase fused to the ligand binding domain of the human estrogen receptor, allow locally triggering mutations after tamoxifen injections [167]. Extending this to TRs or D2 will allow spatio-temporally controlling their mutations, triggering them at adult stages in the tissues of interest such as BAT, WAT muscle and brain. In this way, it will be possible to extract from the observations the very essence of T3 role in adaptive thermogenesis in each of the concerned tissues.

### 6.2. Potential Therapeutical Applications: The Hope Raised by a New Class of Compounds

Given the beneficial metabolic effects of T3, including lowering serum cholesterol and its potentiation of energy expenditure, T3 has been considered to be a potential drug target to fight metabolic diseases. However, its use is precluded by its advert effects, particularly in heart. A lot of efforts have been dedicated to design molecules to target TRβ and more precisely its liver action to fight hypercholesterolemia, because TRβ seems to have only a minor action in heart. Several TRβ agonists have been obtained, efficient to decrease serum cholesterol but all failed in clinical trials due to off target effects, as previously reviewed [168]. 

A breakthrough has been made by coupling T3 to incretins to target it to specific cell types [169]. The incretins are small peptides that bind to specific transmembrane receptors, allowing them to be selectively targeted to the cells expressing the receptor. After reaching the cells, incretins trigger a cascade of phosphorylation intracellularly [170]. 

This principle has been applied with glucagon coupled to T3 (Glc-T3 compound) [171]. This compound mainly targets the liver and in a lesser extent the WAT, due to the expression pattern of the glucagon receptor [172]. By doing so, it lowers cholesterol, increases fatty acid oxidation, and triggers WAT browning. Short term treatment protects from atherosclerosis, nonalcoholic steatohepatitis, and limits obesity in mice models of these diseases. Synchronized signaling driven by glucagon and T3 reciprocally minimizes the inherent harmful effects of each hormone. Indeed, liver-directed T3 action offsets the diabetogenic liability of glucagon, and glucagon-mediated delivery spares the cardiovascular system from adverse T3 action. To date, that is the best compound using selective T3 activity. 

Taking advantage of this concept targeting T3 to BAT or hypothalamus would be of a great interest in the context of thermogenesis, provided to find incretin receptors specifically expressed in these tissues. In this way, metabolic benefits of T3-mediated thermogenesis could be reached while bypassing the undesired effects of thyroid hormone administration [5,6,7].

## 7. Conclusions

The discovery of Lopez et al. concerning the central mode action of T3 [8] that could recapitulate some of its metabolic effects called into question what was previously thought. However, looking at the general picture, it appears that local should not be opposed to central, as the two modes of action seem to complement one another. The general effect observed under hyperthyroidism effect is most likely a combination of both. With the progresses made in generating specific mice model, including inducible tissue-selective mutations, new evidence should emerge shortly to clarify the debate. Meanwhile, the precise molecular mechanisms by which T3 regulates adaptive thermogenesis remain unclear and the emergence of these innovative mice models should help to elucidate the situation. This is of a particular interest as thyroid hormones or their agonists harbor a great metabolic potential when freed of their adverse effects. Understanding how and in which tissues thyroid hormones act should enable identifying new levers to potentiate their metabolic effects and take part in the fight against obesity and metabolic disorders.

Although the transgenic murine models and their future improvement are the cornerstone to decipher T3-mediated effects on adaptive thermogenesis, it must be remembered that these data should be carefully extrapolated to humans. Indeed, there are significant differences in the physiological regulation of thermal homeostasis in the two species. Notably, wearing clothes and living in heat regulated house keep humans closer to thermoneutrality. In sharp contrast, most of the evidence derived from mice have been obtained at room temperature, below thermoneutrality. In this respect, one way to “humanize” mice thermal homeostasis would be to systematically perform mice experiments at thermoneutrality. In this way, the observations would be closer to human physiology and more likely to participate in developing human strategies to counter obesity through adaptive thermogenesis.
